# Post-Graduate Study in Medicine.—I

**Published:** 1912-06-01

**Authors:** 


### POST-GRADUATE STUDY IN MEDICINE.—I.

### The Continental and English Systems of Study Compared.

THE truism that the medical man is always a student has been emphasised from very early times by almost all practitioners who have stood in the front rank of their contemporaries. The learned Boerhaave, after nearly forty years of practice, wrote that he was busily engaged in studying the details of a new method, and if anyone possessed of a knowledge of medical history wished to multiply instances of a similar nature, where men, already expert in one or more branches of their profession, had set themselves the task of learning something new, there would be no difficulty in finding many such cases.

### SOME FAMOUS POST-GRADUATE STUDENTS.

Those who delight to marshall the antique in support of their argument can adduce the Leipzig and Ebers papyri and codices, the remnants of Rhazes, and the urological monograph of Trotula, the sapient lady doctor of Salern. But it is scarcely necessary to buttress the statement with the weight of authoritative pronouncements by ancient and modern masters. Anyone familiar with the trend of medical science and with the recent advances which have been made not only in medicine itself, but in the collateral arts and sciences, must admit that it is impossible for the medical curriculum to embrace all that is necessary for the proper instruction of the student. The constant progress of science requires an equally constant effort on the part of the medical man towards furthering, extending, and, so far as is possible for him, completing his study. Such an effort can only be successful if it is made earnestly and thoroughly, and the ideal of post-graduate instruction in medicine should therefore be to furnish the practitioner with the means and the opportunities to enable him to be successful in his endeavour.

Post-graduate instruction in medicine, it may be argued, is a matter that concerns the medical profession, the faculties of the Universities, and the Councils of the Colleges, and that the public has nothing to do with it, no interest in it, and no responsibilities with regard to it. Such an attitude, however, is indefensible on the part of one who takes an interest in national well-being and is conscious of the fact that, in present circumstances, the medical profession has important relations to the nation. The better educated its individual members are, the better equipped with knowledge, both practical and theoretical, the better will they be able to fulfil their duties and to render those important national services which no other profession can equal. The vital importance of this fact, that the education of the medical practitioner is not merely a question for faculties and colleges, has been well recognised in Germany. It has even been recognised in Russia, where post-graduate instruction in medicine is cared for by the State, where post-graduate institutions fully equal to those that exist in America and Italy, and superior to any that we possess in this country, are in active working, supported by State contributions and to some extent controlled by ministerial departments.

In this country no organised effort has as yet been made to come into line with the advances of these two countries, and a glance at the state of affairs in Germany will readily show how far behind her neighbour England stands in this matter of post-graduate instruction. The main points of the Berlin programme are easily summarised. The State and the profession have joined hands and formed the Central Committee for Post-Graduate Instruction in Medicine (*Das Zentral Komitee fuer das aerztliche Fortbildungswesen*). This important organisation, under whose immediate control nearly all the post-graduate courses in the Empire are now carried on, was formed in 1900 as the result of a widespread feeling on the part of the most prominent medical men that systematic and organised effort was necessary in the interests of the public, no less than in those of the medical profession, to establish a series of efficient courses for the benefit of general practitioners.

Prior to the formation of the Central Committee private committees and organisations had dealt with the matter, and the late Empress Frederic had prominently interested herself in the movement, but the State, as such, had not allied itself with the profession. Once formed, however, the Central Committee set to work to organise graduate instruction in Germany, and during the ten years that it has been in existence it has achieved results which we in this country can only envy and wonder at.

### THE POST-GRADUATE COMMITTEE IN BERLIN.

It has interested the public, secured the cordial co-operation of the State, vanquished the lukewarmness of the profession itself, and made its courses conspicuous successes, which serve as examples to any school that ventures to establish similar classes. It has built, endowed, and equipped the magnificent Kaiserin Friedrich Haus in the Luizenplatz at Berlin, the centre of post-graduate work for the world and the palace of the post-graduate student; it has formed academies, which are post-graduate universities in the best sense of the word, at centres where, although the clinical material is enormous, as at Cologne and Dusseldorf, no clinical teaching has hitherto existed; it has organised regular annual courses in no less than 48 cities in the Empire which are attended by practitioners from all parts of the surrounding districts, and it has once and for all put the post-graduate study movement in Germany on a sure footing. This has only been achieved by strenuous hard work and by the persevering activity, which in many cases meant perpetual self-sacrifice, on the part of the leaders of the profession, and the loyal, energetic, and cordial co-operation of the State. Chief among the many who have worked so earnestly and successfully for the benefit of the profession and of the nation are Professor von Waldeyer and the general secretary of the Kaiserin Friedrich Haus, Professor Dr. Robert Kutner, C.V.O., whose name is well known to all those interested in the movement. Through Professor Kutner's initiative the magnificent institution which is the head office of the Committee was started at Berlin, and it is mainly owing to his unwearied effort on behalf of post-graduate study that the recently appointed International Committee has been formed.

THE IDEAL OF POST-GRADUATE STUDY.

The ideals which the German Committee has held in view are exceedingly simple and eminently practical. It is the general practitioner engaged in active professional work who requires help and teaching.

Every class or course should be so modelled that it is available for him, no matter how poor his means are or how limited his time. For these reasons the courses should be both practical and theoretical, comprising all subjects in which he is likely to be interested and which are calculated to further his knowledge. Furthermore, they should be free, given at such times as are most convenient for him to attend, and at places which are close to his home address, while finally they should be classes of limited membership, restricted to qualified practitioners. All the German courses are of this nature. The practitioner pays a small subscription, just sufficient to cover clerical expenses, and is then at liberty to attend all the courses given under the auspices of the Central Committee.

*(To be continued.)*

